# Hypoxic lung adenocarcinoma‐derived exosomal miR‐1290 induces M2 macrophage polarization by targeting SOCS3

**DOI:** 10.1002/cam4.5954

**Published:** 2023-04-20

**Authors:** Jiahui Gu, Shengrui Yang, Xueying Wang, Yining Wu, Jia Wei, Jian Xu

**Affiliations:** ^1^ Department of Laboratory Medicine the First Affiliated Hospital of Nanjing Medical University Nanjing China; ^2^ Branch of National Clinical Research Center for Laboratory Medicine Nanjing China

**Keywords:** exosome, hypoxia, lung adenocarcinoma, macrophage polarization, miR‐1290

## Abstract

**Background:**

Exosomes are critical mediators of tumor cell‐microenvironment cross talk. However, the mechanisms by which hypoxic Lung adenocarcinoma (LUAD)‐derived exosomes modulate macrophage polarization remain largely unknown. The aim of this study was to investigate the effects of hypoxic LUAD‐derived exosome on macrophage polarization and explore the underlying molecular mechanism.

**Materials and methods:**

LUAD‐derived exosomes were isolated, and then confirmed by transmission electron microscopy, nanoparticle tracking analysis, and Western blot. Internalization of exosomes in macrophages was detected by confocal microscope. Gain‐ and loss‐of‐function experiments, rescue experiments, and xenograft models were performed to uncover the underlying mechanisms of exosomal miR‐1290 induced macrophage polarization in vitro and in vivo.

**Results:**

miR‐1290 was enriched in hypoxic LUAD cancer cell‐derived exosomes and could be transferred to macrophages. Overexpression of miR‐1290 in macrophages‐induced polarization of M2 phenotype. Luciferase assay verified SOCS3 was the target of miR‐1290. Hypoxic LUAD cell‐derived exosomal miR‐1290 activated the STAT3 signaling pathway by targeting SOCS3 to promote M2 macrophage polarization.

**Conclusion:**

Hypoxic LUAD cells generate miR‐1290‐rich exosomes that promote M2 polarization of macrophages. Targeting exosomal miR‐1290 may provide a potential immunotherapeutic strategy for LUAD.

## Graphical abstract

MiR‐1290 was enriched in hypoxic LUAD cancer cell‐derived exosomes and could be transferred to macrophages. Furtherly, miR‐1290 induced macrophage M2 polarization by activating the SOCS3/STAT3 pathway, which promoted LUAD progression.

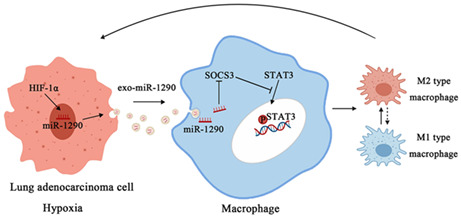

## 1 INTRODUCTION

Lung cancer is one of the most common malignant tumors and the leading cause of cancer‐related mortality worldwide.^1^ Lung adenocarcinoma (LUAD) is the most prevalent subtype, with an incidence of approximately 40% among all lung cancer cases.^2^ The average five‐year survival rate for patients with lung cancer is as low as 15 percent.^3^ Recently, cancer immunotherapy has shown a promising effect in a variety of cancer types, including LUAD.^4^, ^5^

Macrophages are the most abundant infiltrative immune cells in the tumor microenvironment (TME).^6^ Tumor‐associated macrophages (TAMs) in the TME have been suggested as a new target for cancer immunotherapy.^7^ TAMs display diverse phenotypes and functions in response to different TME signals. They can be polarized into classically (M1) or alternatively (M2) activated cells. M1 macrophages exert a pro‐inflammatory and antitumor function, whereas M2 macrophages play a supportive role in cancer by interfering with adaptive immunity and the function of inflammatory pathways.^8^

Macrophages are abundant in lung cancer tissues. A large number of M2‐type macrophages are significantly correlated with worse prognosis in lung cancer patients.^9^ Previous studies have suggested that modulating the phenotype of intratumoral macrophages can dramatically augment the effect of immunotherapy for lung cancer.^10^ However, the potential mechanisms of macrophage polarization in LUAD remain largely unknown.

Exosomes act as the key mediator of intercellular cross talk in the TME.^11^ Exosomes can be secreted by tumor cells and then migrate from their primary positions to transfer functional molecules into recipient cells, thereby modulating the TME to promote tumor development.^12^ Tumor‐derived exosomes can also carry a variety of immunosuppressive signals, which invalidate antitumor immune effectors and promote tumor immune evasion.^13^

Hypoxia, a common feature of the TME, can promote the release of exosomes from cancer cells.^14^ Hypoxic cancer‐derived exosomes can remodel the TME, promote cancer development, and enhance angiogenesis and premetastatic niche formation.^15^ Park et al.^16^ reported that let‐7a was selectively packed into exosomes under hypoxia and that exosomal let‐7a enhanced the M2‐like polarization of TAMs to evade host immunity.

In this study, we demonstrate that hypoxic LUAD cell‐derived exosomes overexpressing miR‐1290 can polarize M2 macrophages by targeting SOCS3, which further promotes LUAD progression. These findings suggest that miR‐1290 is a potential target for macrophage repolarization in LUAD immunotherapy.

## 2 MATERIALS AND METHODS

### 2.1 Cell culture

The human bronchial epithelial (HBE) cell line, human LUAD cell lines A549 and H1299, and human peripheral blood monocyte line THP‐1 were purchased from the Cell Bank of the Chinese Academy of Sciences. HBE, H1299, and THP‐1 cells were maintained at 37°C in a 5% CO_2_ humidified atmosphere in complete RPMI 1640 medium (HyClone) supplemented with 10% fetal bovine serum (FBS; Gibco) and 1% penicillin and streptomycin (Ruiyang). A549 cells were cultured at 37°C in a 5% CO_2_ humidified atmosphere in complete DMEM with high glucose (HyClone). For hypoxic treatment, the cells were cultured in a hypoxic cell incubator with 1% O_2_.

### 2.2 Macrophage induction from monocytes

To induce differentiation into macrophages, THP‐1 cells were stimulated with 100 ng/mL phorbol‐12‐myristate‐13‐acetate (PMA; Sigma) for 24 h, and next maintained in refreshed medium without PMA for 3 days prior to use.

### 2.3 Cell transfection

miR‐1290 mimic and miR‐1290 inhibitor were generated by Ruibo. The overexpression plasmids pCDNA3.1‐SOCS3 and pCDNA3.1‐HIF‐1α and HIF‐1α siRNAs were constructed by GenePhama.

Cell transfection was performed using Lipofectamine™ 3000 transfection reagent (Invitrogen) according to the manufacturer's protocol. All cells were cultured in complete medium for at least 24 h and rinsed with phosphate‐buffered saline (PBS, pH 7.4) before transfection.

### 2.4 Exosome isolation and identification

The cell lines were cultured in normal medium until they reached 70% confluence, and thereafter, the medium was replaced with DMEM or RPMI 1640 with 2% exosome‐depleted FBS. Then, the cell culture medium was harvested after 24–48 h and centrifuged at 4°C for 20 min at 3000 g. The cell culture supernatant was collected into an ultrafiltration tube and centrifuged at 4°C for 30 min at 1000 g. The concentrated supernatant was filtered through a 0.22 μm filter, followed by addition of exosome extraction reagent (SBI). The sample was mixed evenly, incubated overnight, and then centrifuged at 1500 × g for 30 min. The pelleted exosomes were resuspended in PBS and stored at −80°C until further use.

The size of exosomes was analyzed using a Nanosight nanoparticle tracer instrument (Malvin instruments). Exosomes were examined via transmission electron microscopy (TEM, Tecnai G2 F20, FEI). Exosomes were suspended in 2.5% glutaraldehyde for 2 h and washed with PBS. Subsequently, 20 μL of exosome suspension was dropped onto a small carbon‐coated copper grid (Electron Microscopy Sciences), and excess suspension was removed with filter paper. For exosome staining, a total of 3% phosphotungstic acid was applied to the grid for 1 min. After drying, TEM was used to observe the morphology of the stained exosomes.

### 2.5 Exosome labeling and tracking

Purified exosomes were labeled with PKH26 red fluorescent membrane linker dye (Sigma–Aldrich) according to the manufacturer's instructions. After removal of excess dye, the labeled exosome pellets were resuspended and added to the cultured macrophages for the studies of exosome uptake. After incubation for 24 h and two washes with PBS, the cells were fixed with 4% paraformaldehyde, stained with DAPI to visualize nuclei and immediately observed by laser confocal microscopy (Zeiss).

### 2.6 Luciferase assays

HEK‐293 T cells were cotransfected with the reporter plasmid containing wild‐type or mutant SOCS3 3'‐UTR (GenePharma) and miR‐1290 mimic or control using Lipofectamine™ 3000 (Invitrogen). Cell lysates were collected 24 h after transfection, and next, firefly and Renilla luciferase activities were assessed using a Dual‐Luciferase® Reporter Assay kit (Promega) in accordance with to the manufacturer's instructions. Each set of experiments was performed in triplicate.

### 2.7 Flow cytometry

To analyze macrophage surface markers, cells were incubated with primary antibody against a specific antigen. The following antibodies were used: allophycocyanin (APC)‐labeled antihuman CD14 antibody was obtained from BD Biosciences; phycoerythrin (PE)‐labeled antihuman CD80 and CD163 antibodies and Alexa Fluor‐labeled antihuman CD206 antibody were obtained from Biolegend. Flow cytometric data were recorded with an Accuri C6 flow cytometer (BD Biosciences) and processed using CellQuest software (BD Biosciences).

### 2.8 RNA extraction and real‐time quantitative PCR

Total RNA was extracted with TRIzol reagent (Invitrogen), and complementary DNA was synthesized using a reverse transcription system (Vazyme Biotech) following the manufacturer's protocol. Real‐time quantitative PCR (qPCR) was performed with SYBR Color qPCR Master Mix (Vazyme Biotech) on a 7500 Real‐time PCR System (Applied Biosystems). The primers used are shown in *Supporting Information* Table S1. For endogenous control, β‐actin was used. Relative expression values were calculated by 2^−ΔΔCt^.

### 2.9 Western blot analysis

Cell or exosome lysis was performed in RIPA lysis buffer with complete proteolytic and phosphatase inhibitors (Sigma). A total of 40 μg of protein per well was loaded onto SDS‐polyacrylamide gels and next transferred onto polyvinylidene difluoride membranes (PVDF; Millipore). Thereafter, membranes were blocked with 5% skimmed dry milk and then incubated with primary antibodies overnight at 4°C, followed by incubation with horseradish peroxidase‐conjugated secondary antibody for 1 h at room temperature. Finally, the membranes were analyzed using an automatic chemiluminescence imaging analysis system (Tanon). The primary antibodies anti‐CD9 (CST), anti‐CD63 (Abcam), anti‐SOCS3 (R&D systems), anti‐HIF1α (CST), anti‐p‐AKT (CST), anti‐AKT (CST), anti‐p‐STAT3 (CST), anti‐STAT3 (CST), and anti‐GAPDH (Proteintech) were used in the experiments.

### 2.10 Cell proliferation assay

A549 and H1299 cells were seeded in 96‐well culture plates at 3000 cells per well in triplicate. On the next day, the cells were cultured with the supernatant of PMA‐induced THP‐1 cells treated with hypoxic or normoxic exosomes. After culture for 0 h, 24 h, 48 h, or 72 h, 10 μL of Cell Counting Kit‐8 (CCK‐8) reagent (Beyotime) was added and incubated with the cells for 1 h at 37°C. The absorbance was read at 450 nm, and growth curves were constructed on the basis of the OD values.

### 2.11 Cell scratch assay

Cells were seeded in 6‐well plates at a density of 1 × 10^6^ cells per well and allowed to incubate for 24 h. After that, a straight line was scraped with a sterile pipette tip. Detached cells were removed. Then, the cells were incubated for 24 h after scratching. The area of the scratches was measured using ImagePro Plus software.

### 2.12 Cell invasion assay

Cells in logarithmic phase were collected and resuspended to a final concentration of 2 × 10^5^ cells/mL. Then, 100 μL of cell suspension was added to Matrigel‐coated transwell inserts, and 500 μL of precooled DMEM containing 10% FBS was put under the inserts. After incubation for 24 h in 5% CO_2_ at 37°C, cells in the inserts were swabbed, while invaded cells from the chamber were fixed with methanol for 30 min and stained with 0.1% crystal violet for 15 min. Cells were observed and photographed under an inverted microscope. Ten fields were randomly selected for cell counting.

### 2.13 Animal model

Animal experiments were approved by the First Affiliated Hospital of Nanjing Medical University. Four‐week‐old male nude mice were provided by GemPharmatech. PMA‐induced THP‐1 cells were incubated for 24 h with exosomes derived from A549 cells treated with hypoxia, normoxia, and miR‐1290 mimic or inhibitor. Then, the conditioned macrophages were mixed with equivalent amounts of A549 cells. This cell mixture (1 × 10^6^) was injected subcutaneously into the axilla of nude mice. Tumor volumes were examined with a caliper every 7 days and calculated using the formula 0.5 × length × width^2^. A subcutaneous graft curve was drawn with the tumor growth time (d) as the abscissa and the volume mean (mm^3^) as the ordinate. After 42 days, the mice were sacrificed, and the xenograft tumors were excised for weight measurement and processed for immunohistochemistry analysis.

### 2.14 Immunohistochemistry (IHC)

IHC staining was conducted according to the manufacturer's protocol (Maixin Bioengineering). In brief, tissue samples were fixed, paraffin‐embedded, dewaxed, rehydrated, and subjected to antigen retrieval. Then, the samples were stained with antihuman CD163, Ki67, or CD31 antibody (Abcam) at 4°C overnight, followed by incubation with HRP‐labeled secondary antibodies for 30 min at 37°C. Finally, sections were incubated with DAB solution (Maixin Bioengineering) and counterstained with hematoxylin. The results were scanned using a digital pathology slice scanner (Motic), and the number of positive cells was counted in at least 10 independent high‐power fields.

### 2.15 Statistical analysis

All data were processed using SPSS 24.0 software (SPSS Inc.). The mean ± standard deviation is used to express the measurement data. Comparisons between two groups were performed using Student's *t*‐test or a nonparametric Mann–Whitney U test. All in vitro experiments were repeated at least three times. *p* values <0.05 were considered significant and are denoted by “*”; *p* values <0.01 are denoted by “**”. *p* > 0.05 was considered not significant and is denoted by “NS”.

## 3 RESULTS

### 3.1 Hypoxic lung adenocarcinoma cell‐derived exosomes increase M2 polarization

**FIGURE 1:**
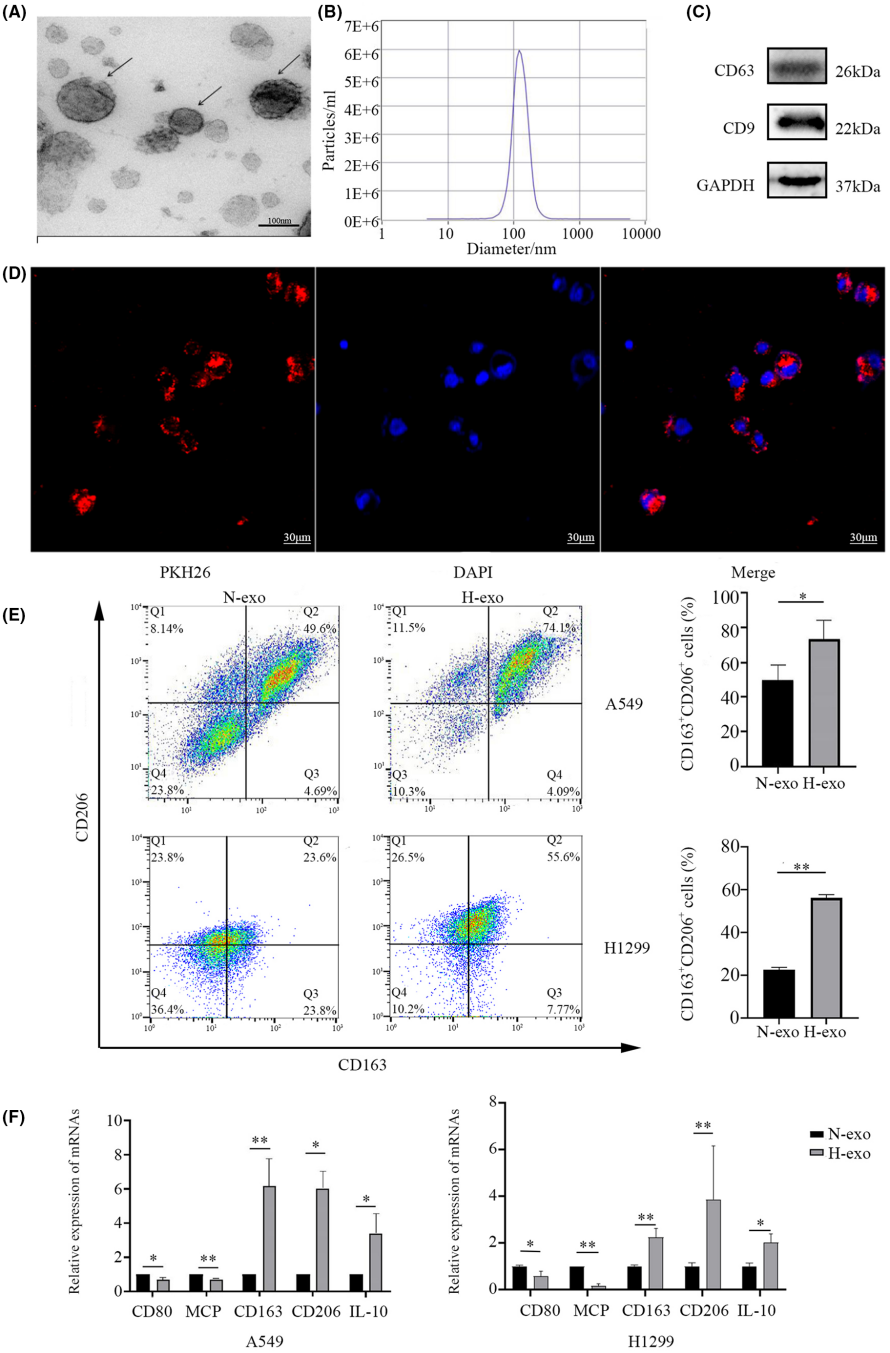
Exosomes secreted from hypoxic lung adenocarcinoma cells increase M2 polarization. (A) TEM images of exosomes isolated from the medium of the A549 LUAD cell line. (B) NTA of A549 exosomes. (C) Western blot analysis of the exosomal marker proteins CD9 and CD63. (D) Phagocytosis of PKH26‐labeled A549 cell‐derived exosomes by macrophages (magnification: 200×). (E) Flow cytometry was used to detect the expression of M2 macrophage markers (CD163 and CD206) in THP‐1 cells treated with A549 exosomes under normoxia and hypoxia. (F) qRT‐PCR was used to detect the mRNA levels of M1 macrophage markers (CD80 and MCP‐1) and M2 macrophage markers (CD163, CD206, and IL‐10) in THP‐1 cells treated with A549 exosomes under normoxia and hypoxia. *p < 0.05; **p < 0.01. N‐, normoxia; H‐, hypoxia.

To investigate the role of exosomes from LUAD cells in macrophage polarization under hypoxic conditions, exosomes were isolated for identification. Transmission electron microscopy (TEM) showed a disk shape of exosomes (Figure 1A). Nanoparticle tracking analysis (NTA) showed that the diameter of exosomes was approximately 120 nm (Figure 1B). Furthermore, western blotting confirmed that the exosomal membrane protein markers CD9 and CD63 were highly expressed (Figure 1C). Next, we examined the interaction between exosomes and macrophages. After coculture of THP‐1 macrophages with PKH26‐labeled exosomes, fine fluorescent particles around the cell nucleus were observed via laser co microscopy (Figure 1D), confirming that macrophages can engulf and internalize LUAD‐derived exosomes.

To examine the impact of hypoxic LUAD exosomes on macrophage polarization, exosomes from A549 and H1299 cells under hypoxia and normoxia were separately cocultured with THP‐1 cells. Flow cytometry was used to detect the expression of the M2 markers CD163 and CD206. The expression levels of these M2‐type markers in the hypoxia‐treated group were significantly higher than those in the normoxia‐treated group (Figure 1E). qPCR was used to detect the mRNA levels of M1 macrophage markers (CD80 and MCP‐1) and M2 markers (CD163, CD206, and IL‐10). Consistently, hypoxia treatment strikingly promoted the expression of M2‐type genes and inhibited the expression of M1‐type genes (Figure 1F). Hence, the results showed that hypoxia‐treated LUAD cell exosomes can promote macrophage M2‐type polarization.

### 3.2 M2 macrophages polarized by hypoxic exosomes elicit a tumor‐promoting phenotype

**FIGURE 2:**
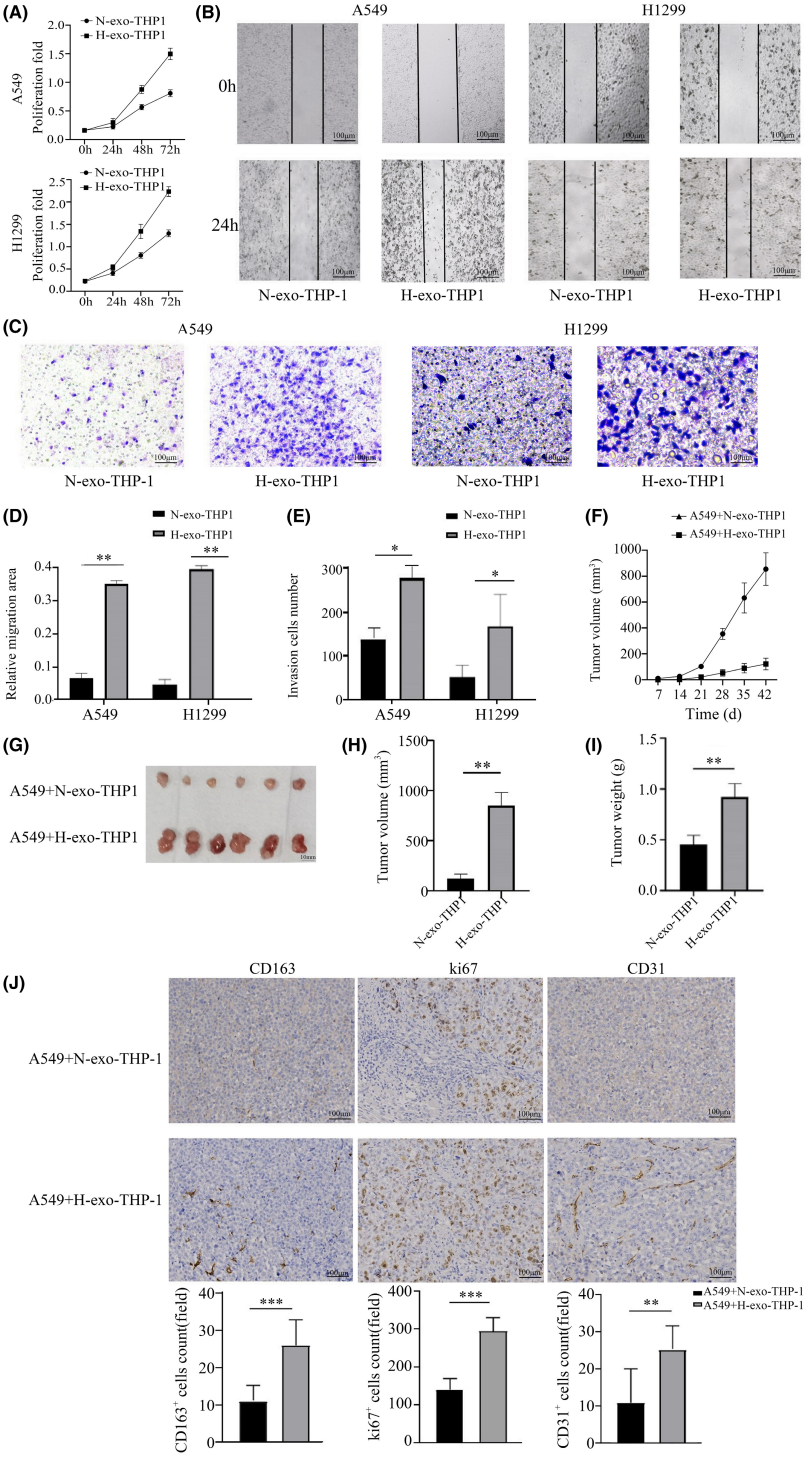
M2 macrophages polarized by hypoxic LUAD exosomes elicit a tumor‐promoting phenotype. (A) CCK8 assay of LUAD cells incubated with supernatants from macrophages treated with normoxic or hypoxic exosomes. (B, D) Wound healing assay of LUAD cells incubated with supernatants from macrophages treated with normoxic or hypoxic exosomes. Representative images (magnification, ×40) and quantifications are shown. (C, E) The invasive capacity of LUAD cells cocultured with exosome‐treated THP‐1 macrophages was determined using an in vitro transwell coculture system. Representative images of invaded cells (magnification, ×40) and quantifications are shown. (F) Subcutaneous tumor volume in nude mice was monitored weekly after injection with A549 cells and exosome‐treated THP‐1 macrophages. (G) Images of excised tumors from nude mice on Day 42 after cell injection. (H, I) Quantification of xenograft tumor volume and weight on Day 42 after cell injection. (J) Immunohistochemistry analysis of Ki67, CD31, and CD163 staining was carried out on xenograft tumor sections. Representative staining is shown (magnification, ×40). *p < 0.05; **p < 0.01. N‐, normoxia; H‐, hypoxia.

To explore whether M2 macrophages educated by hypoxic LUAD exosomes could exert tumor‐promoting functions, we collected the supernatant of THP‐1 macrophages treated with exosomes derived from hypoxic or normoxic LUAD cells. This supernatant medium was then used to treat A549 or H1299 cells, and a CCK8 assay was performed to assess LUAD cell proliferation. As shown in Figure 2A, the supernatant medium of macrophages treated with hypoxic exosomes enhanced the proliferation of both A549 and H1299 cells. Furthermore, cell migration and invasion were investigated using cell scratch and transwell assays. As shown in Figure 2B–E, macrophages treated with hypoxic exosomes promoted A549 and H1299 cell migration and invasion.

To further validate that macrophages educated by hypoxic LUAD exosomes could promote the progression of LUAD in vivo, A549 cells mixed with conditioned macrophages stimulated by hypoxic or normoxic exosomes were subcutaneously injected into nude mice (*n* = 6). Subcutaneous tumor volumes were examined weekly with calipers, and the growth curve was analyzed (Figure 2F). After 6 weeks, we found that mice treated with hypoxic exosomes had larger tumors than those treated with normoxic exosomes (*p* < 0.01, Figure 2G,H). Moreover, the tumors in the hypoxic exosome‐treated group were significantly heavier than those in the normoxic exosome‐treated group (*p* < 0.01, Figure 2I). We utilized IHC staining to detect the expression of the tumor proliferation marker Ki67, the microangiogenesis marker CD31 and the M2 macrophage marker CD163 in tumor tissues. As shown in Figure 2J, the expression of CD163 was significantly increased in the tumor tissue of the hypoxia‐induced group, suggesting increased infiltration of M2‐type macrophages. Meanwhile, the expression of Ki67 and CD31 was also strongly enhanced in the hypoxia‐induced group, which was consistent with the changes in tumor volume and mass, suggesting tumor‐promoting effects of M2 macrophages. Taken together, these results suggest that hypoxic LUAD exosomes can induce M2‐type macrophage polarization, which promotes tumor progression.

### 3.3 miR‐1290 is highly expressed in exosomes derived from hypoxic LUAD cells and can be transferred to macrophages through exosomes

**FIGURE 3:**
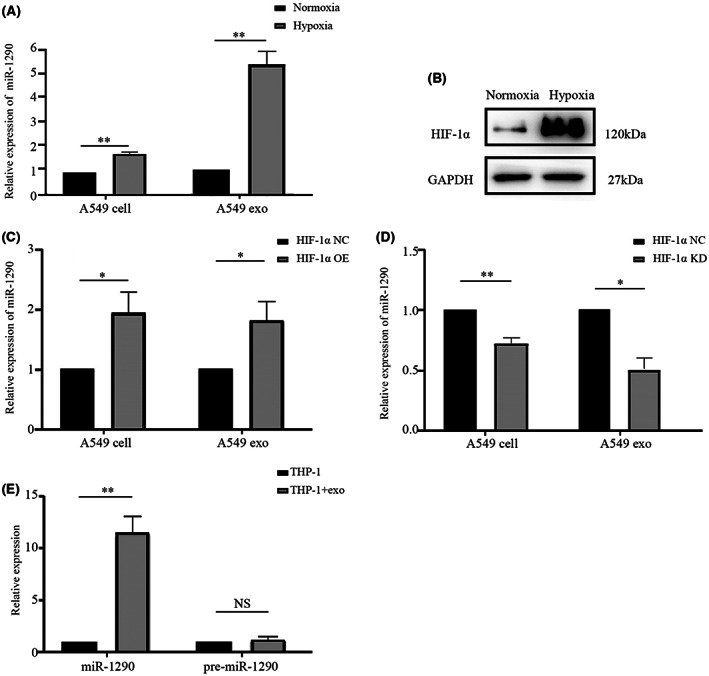
miR‐1290 is highly expressed in exosomes derived from hypoxic LUAD cells and can be transferred to macrophages through exosomes. (A) The levels of miR‐1290 in hypoxic and normoxic A549 cells and exosomes. (B) Hypoxia increased HIF‐1α expression in A549 cells. (C) The expression of cellular and exosomal miR‐1290 in HIF‐1α overexpressing A549 cells under normoxic conditions. (D) The expression of cellular and exosomal miR‐1290 in HIF‐1α knockdown A549 cells under hypoxic conditions. (E) The levels of mature and pre‐miR‐1290 in THP‐1 cells treated with hypoxic A549‐derived exosomes. *p < 0.05; **p < 0.01.

Our previous study showed that miR‐1290 was highly expressed in LUAD tissues and serum and that exosomal miR‐1290 could be a potential biomarker for LUAD adjuvant diagnosis and prognosis prediction.^17^ To elucidate whether the miR‐1290 level was increased under hypoxia, we detected the expression of miR‐1290 in intracellular and exosomal compartments of LUAD cells. The results showed that both intracellular and exosomal miR‐1290 were markedly elevated in A549 cells under hypoxia (Figure 3A).

HIF‐1α is a critical regulator of the hypoxic adaptive response. As shown in Figure 3B, the expression of HIF‐1α was significantly upregulated in hypoxia‐treated A549 cells. To determine whether the hypoxia‐induced increase in miR‐1290 depends on HIF‐1α, we manipulated HIF‐1α expression in A549 cells. Both cellular and exosomal miR‐1290 expression levels were apparently higher in the HIF‐1α overexpression group (Figure 3C) and lower in the HIF‐1α knockdown group than in the control group (Figure 3D). These results suggest that miR‐1290 expression is dependent on HIF‐1α under hypoxic conditions. Then, we evaluated the change in the expression level of miR‐1290 in THP‐1 cells after treatment with hypoxic A549 exosomes. The level of mature miR‐1290 increased, while the level of pre‐miR‐1290 did not change (Figure 3E). These data illustrate that the increase in miR‐1290 is transferred from hypoxic A549 exosomes.

### 3.4 miR‐1290 induces M2 polarization and a tumor‐promoting phenotype

**FIGURE 4:**
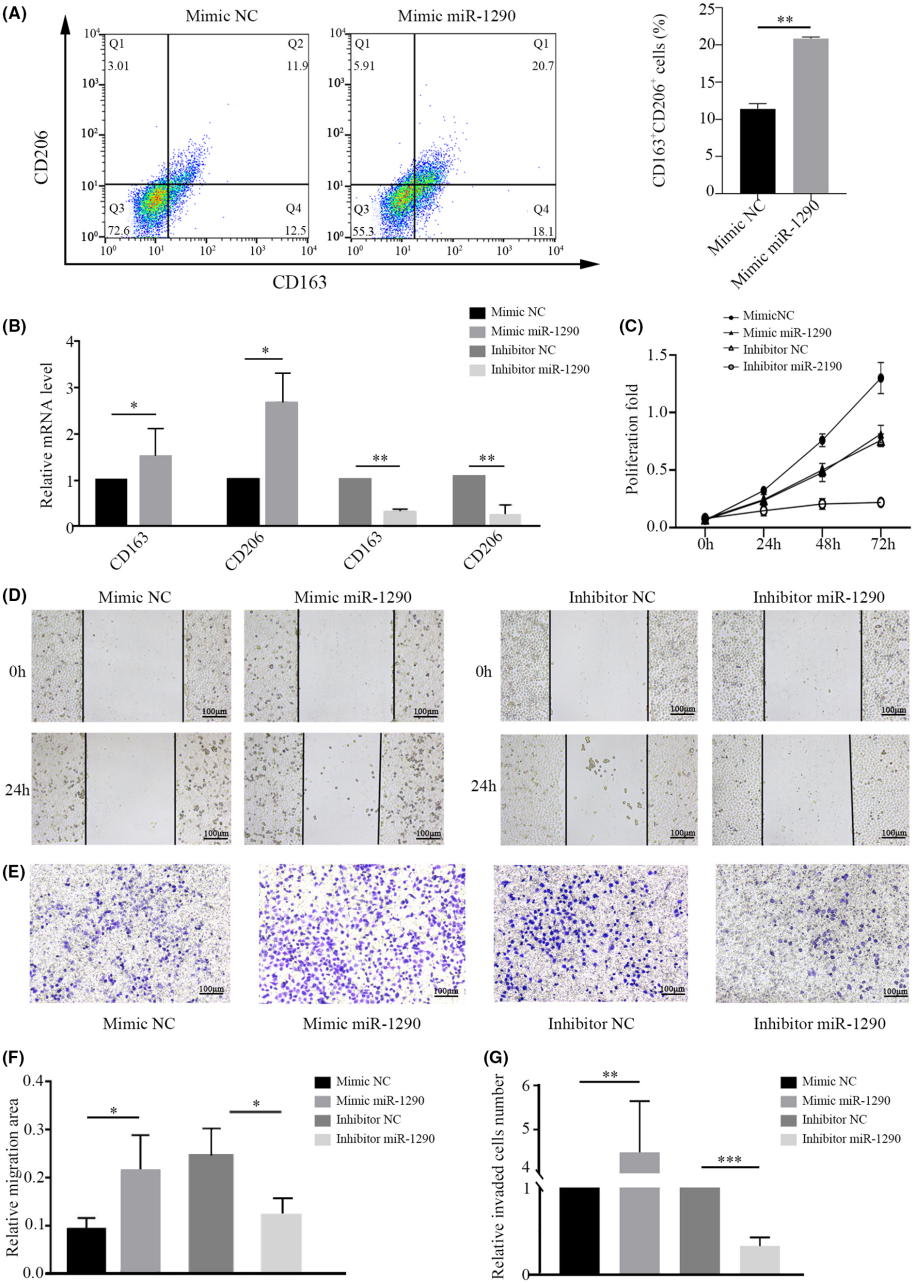
miR‐1290 induces M2 macrophage polarization and enhances a tumor‐promoting phenotype. (A) Flow cytometry analysis of M2 markers in THP‐1 cells transfected with miR‐1290 mimic. (B) RT‐PCR analysis of M2 markers in THP‐1 cells transfected with miR‐1290 mimic or inhibitor. (C) CCK8 assay of A549 cells treated with the supernatants of THP‐1 cells transfected with miR‐1290 mimic or inhibitor. (D) Wound healing assay of A549 cells treated with the supernatants of THP‐1 cells transfected with miR‐1290 mimic or inhibitor (magnification, ×40). (E) Transwell invasion assay of A549 cells cocultured with THP‐1 cells transfected with miR‐1290 mimic or inhibitor (magnification, ×40). (F, G) Quantification of the migration area in the wound healing assay and invaded cells in the transwell invasion assay. *p < 0.05; **p < 0.01.

We next investigated the role of miR‐1290 in macrophage polarization. Flow cytometry showed that miR‐1290 mimic induced an increase in the expression of the M2 markers CD163 and CD206 in THP‐1‐derived macrophages (Figure 4A). Meanwhile, the mRNA levels of M2‐related genes (CD163 and CD206) were strikingly upregulated in THP‐1 cells after transfection with miR‐1290 mimic (Figure 4B).

Then, the supernatant medium of THP‐1 cells transfected with miR‐1290 mimic or inhibitor was used to treat A549 cells. The proliferation, migration and invasion abilities of A549 cells were assessed. CCK‐8 assays demonstrated that A549 cell proliferation increased greatly in the miR‐1290‐mimic group, whereas it decreased dramatically in the inhibitor group (Figure 4C). Further data showed that the migration and invasion abilities of A549 cells were positively regulated by the supernatants of miR‐1290‐overexpressing THP‐1 cells (Figure 4D–G). After miR‐1290 knockdown, the migration and invasion abilities of A549 cells were noticeably attenuated (Figure 4D–G). These results suggest that miR‐1290 can induce M2 polarization, which then enhances the proliferation, migration and invasion of LUAD cells.

### 3.5 miR‐1290 polarizes M2 macrophages by targeting SOCS3 and activating the STAT3 signaling pathway

**FIGURE 5:**
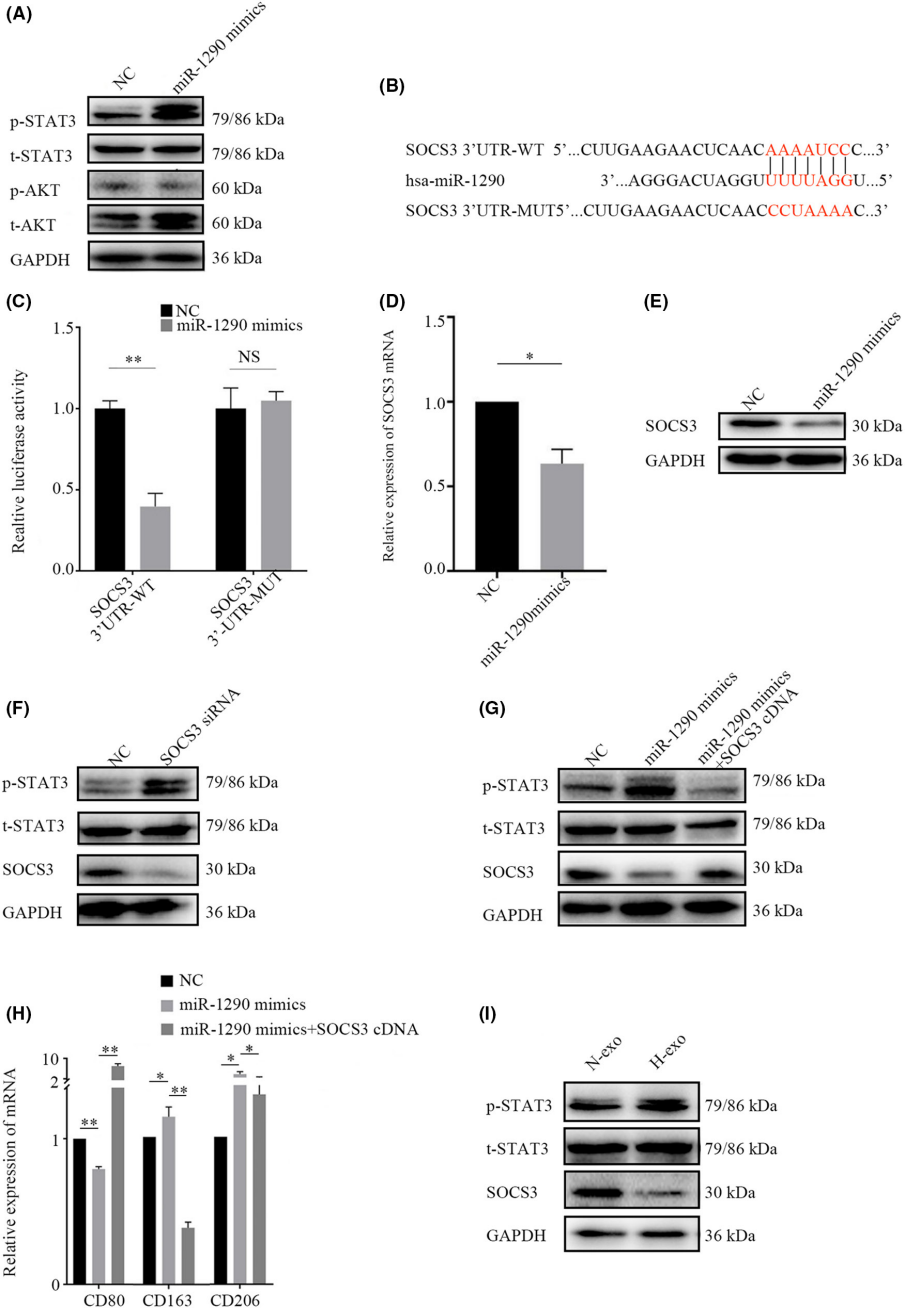
miR‐1290 polarizes M2 macrophages by targeting SOCS3 and activating the STAT3 signaling pathway. (A) The phosphorylation of STAT3 and AKT in THP‐1 cells after miR‐1290 overexpression. (B) Predicted binding sequence of miR‐1290 with the SOCS3 3'UTR according to TargetScan. (C) Luciferase reporter assay in 293 T cells cotransfected with the wild‐type or mutant SOCS3 3' UTR and miR‐1290 mimic. (D) SOCS3 mRNA expression was measured by qRT‐PCR in THP‐1 cells transfected with miR‐1290 mimic. (E) SOCS3 protein expression was examined by western blotting in THP‐1 cells transfected with miR‐1290 mimic. (F) The expression of SOCS3 and phosphorylation of STAT3 in THP‐1 cells transfected with SOCS3 siRNA were examined via western blot analysis. (G) The expression of SOCS3 and phosphorylation of STAT3 in THP‐1 cells cotransfected with miR‐1290 mimic and SOCS3 overexpression plasmid were examined via western blot analysis. (H) The mRNA expression levels of macrophage polarization markers were measured by qRT‐PCR in THP‐1 cells cotransfected with miR‐1290 mimic and SOCS3 overexpression plasmid. (I) The expression of SOCS3 and phosphorylation of STAT3 were examined via western blotting in THP‐1 cells treated with normoxic or hypoxic A549 exosomes. *p < 0.05; **p < 0.01. N‐, normoxia; H‐, hypoxia.

We transfected THP‐1‐derived macrophages with miR‐1290 mimic to explore the underlying mechanisms of miR‐1290 in the induction of M2 macrophage polarization. An analysis of the activation of the classical STAT3 and AKT pathways was performed. Western blot analysis showed that after transfection with miR‐1290 mimic, the phosphorylation level of STAT3 at tyrosine 705 [p‐STAT3 (Tyr705)] was significantly elevated (Figure 5A). Previous studies have reported that M2 polarization is strongly related to the SOCS/STAT pathway.^18^ TargetScan analysis revealed a putative miR‐1290 binding site in the 3'‐UTR of suppressor of cytokine signaling 3 (SOCS3) (Figure 5B), which is a key regulatory gene of macrophage polarization. To verify whether SOCS3 is a target of miR‐1290, 293 T cells were cotransfected with miR‐1290 mimic and luciferase vector with the wild‐type (wt) or mutant (mut) 3'‐UTR of SOCS3. The luciferase activity was greatly reduced in cells cotransfected with miR‐1290 mimic and wt SOCS3 3'‐UTR luciferase reporter gene plasmid; however, no significant inhibition was observed in cells cotransfected with miR‐1290 and mut 3'‐UTR of SOCS3 (Figure 5C). To further confirm that miR‐1290 was able to specifically inhibit SOCS3, we transfected THP‐1 cells with miR‐1290 mimic and measured changes in SOCS3 expression levels. qRT‐PCR and western blotting results showed that overexpression of miR‐1290 significantly inhibited SOCS3 expression (Figure 5D,E).

To investigate whether SOCS3 is responsible for miR‐1290‐induced M2 macrophage polarization, we transfected THP‐1 cells with SOCS3 siRNA and detected the phosphorylation level of STAT3. Western blot analysis demonstrated that knockdown of SOCS3 increased phosphorylated STAT3 levels in THP‐1 cells (Figure 5F). We next cotransfected THP‐1 cells with miR‐1290 mimic and SOCS3 overexpression plasmid and then examined the alterations in the STAT3 pathway and macrophage polarization. The results showed that overexpression of SOCS3 reversed the upregulation of p‐STAT3 induced by miR‐1290 in THP‐1 cells (Figure 5G). Meanwhile, the mRNA levels of the M1 marker CD80 and M2 markers CD163 and CD206 were examined via qRT‐PCR. As shown in Figure 5H, the overexpression of SOCS3 reversed the promoting effect of miR‐1290 on macrophage polarization toward the M2 phenotype. Taken together, these data indicate that miR‐1290 activates the STAT3 signaling pathway by targeting SOCS3 to induce M2 macrophage polarization. Moreover, both the SOCS3 decrease and the p‐STAT3 increase were observed in THP‐1 cells after which were treated with hypoxic A549 exosomes (Figure 5I).

### 3.6 Exosomal miR‐1290 increases M2 polarization and promotes cancer progression in vivo

**FIGURE 6:**
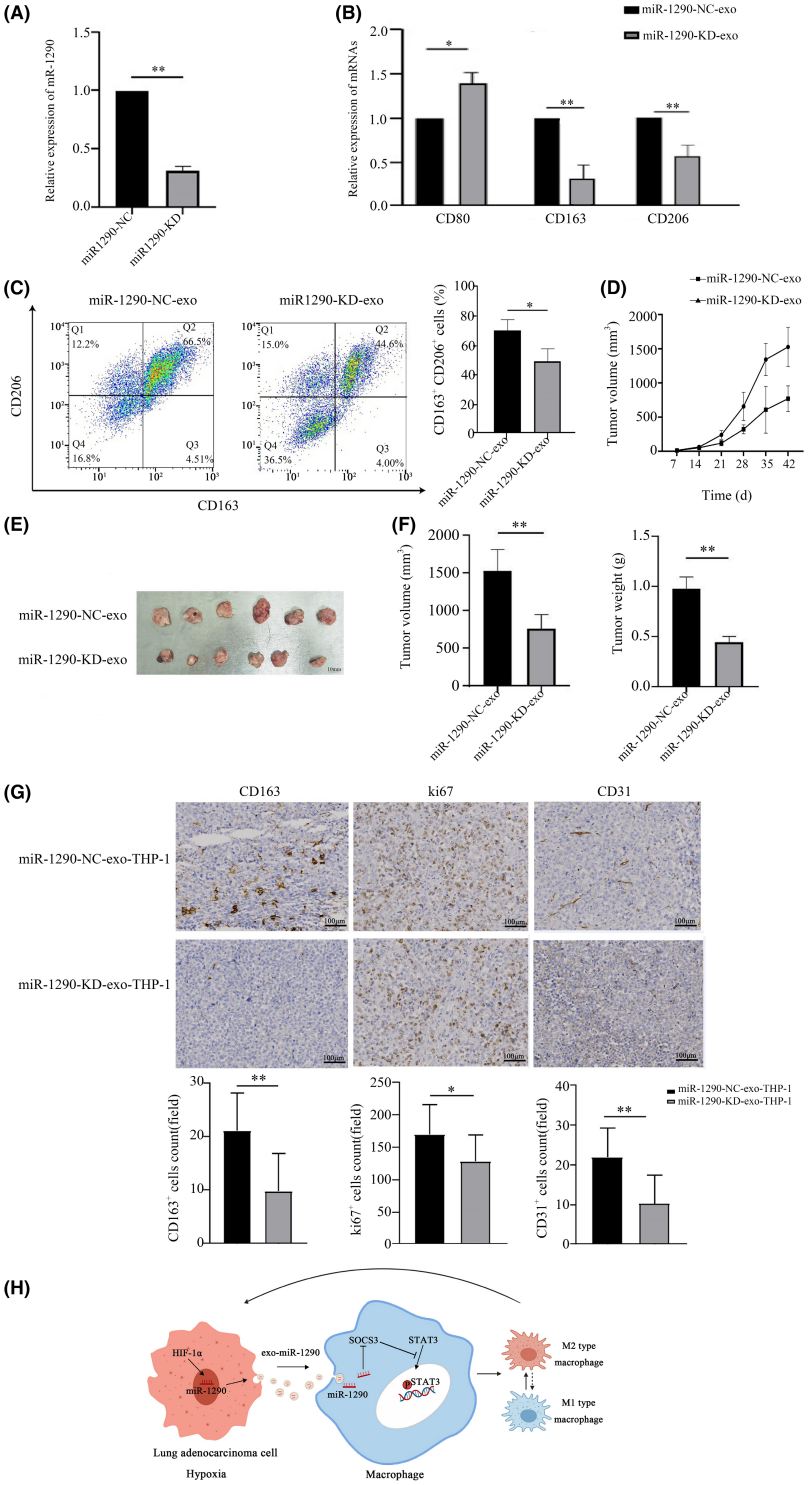
Exosomal miR‐1290 increases M2 polarization and promotes cancer progression. (A) The level of miR‐1290 was measured by qRT‐PCR in A549 cells transfected with miR‐1290‐sponge. (B) The mRNA expression levels of macrophage polarization markers were measured by qRT‐PCR in THP‐1 cells treated with exosomes derived from miR‐1290‐sponge‐transfected A549 cells. (C) Flow cytometry analysis of M2 markers in THP‐1 cells treated with exosomes from miR‐1290‐sponge‐transfected A549 cells. (D) Subcutaneous tumor volume in nude mice was monitored weekly after injection with A549 cells and THP‐1 macrophages treated with exosomes from miR‐1290‐knockdown cells. (E) Images of excised tumors from nude mice on Day 42 after cell injection. (F) Quantification of xenograft tumor volumes and weights on Day 42 after cell injection. (G) Immunohistochemistry analysis of Ki67, CD31, and CD163 staining was carried out on xenograft tumor sections. Representative staining is shown (magnification, ×40). (H) Schematic model illustrating how hypoxic exosomal miR‐1290 promotes M2 polarization. *p < 0.05; **p < 0.01.

First, we established miR‐1290 knockdown in A549 cells (A549‐1290‐KD) by transduction with a lentivirus vector containing the sponge sequences of miR‐1290 (Figure 6A). Next, exosomes derived from hypoxic A549‐1290‐KD cells were added to THP‐1 cells. Macrophage polarization markers were detected by qPCR and flow cytometry. As shown in Figure 6B, miR‐1290‐depleted exosomes (miR‐1290‐KD‐exo) obviously diminished CD163 and CD206 mRNA expression and increased CD80 mRNA expression compared with miR‐1290‐NC‐exo. Moreover, flow cytometry further confirmed this finding (Figure 6C).

To explore the effect of exosomal miR‐1290 on the growth of LUAD in vivo, we established a nude mouse xenograft model. A549 cells mixed with conditioned THP‐1 macrophages stimulated by hypoxic miR‐1290‐KD‐exo or miR‐1290‐NC‐exo were subcutaneously injected into the mice. After six weeks, the miR‐1290‐KD‐exo‐treated group generated smaller tumors than the miR‐1290‐NC‐exo‐treated group (Figure 6D–F). Likewise, the weight of tumors in the miR‐1290‐KD‐exo group was significantly lower than that in the control group (Figure 6F). IHC experiments revealed that the expression levels of CD163, Ki‐67, and CD31 were weaker in the miR‐1290‐KD‐exo group than in the control group (Figure 6G). These results indicate that exosomal miR‐1290 facilitates the growth of LUAD *in vivo*.

## 4 DISCUSSION

Hypoxia is linked to tumor cell proliferation, metastasis, and treatment resistance.^19^ In addition, hypoxia impacts the functions of several immune cells.^20^ Under hypoxic conditions, macrophages are inclined to M2 deviation with protumorigenic effects.^21^, ^22^, ^23^ In the present study, we demonstrated that hypoxic LUAD cells released miR‐1290‐enriched exosomes, which promoted M2 macrophage polarization and further enhanced cancer progression.

Exosomes play an important role in the malignant evolution of cancer. Tumor cells release exosomes that facilitate tumor progression by communicating with the microenvironment and distant organs.^24^ Li et al.^25^ reported that the hypoxic microenvironment stimulated tumor cells to generate miR‐21‐rich exosomes, which were transported to normoxic cells to support prometastatic behaviors. As expected, our results revealed that exosomes could mediate the cross talk between hypoxic LUAD cells and macrophages.

Exosomes have been shown to load noncoding RNAs, which can later be delivered to recipient cells and have effects on cellular function.^26^ Some exosomal miRNAs have been identified as critical regulators of specific cellular and intercellular processes, which are highly prospective targets for cancer diagnosis and treatment.^27^ miR‐19a in exosomes from brain astrocytes was found to be delivered to tumor cells to downregulate PTEN and boost tumor brain metastasis.^28^ miR‐21 can be packaged into exosomes derived from tumor cells, enhance epithelial–mesenchymal transition (EMT),^29^ and induce angiogenesis.^30^, ^31^ Here, we illustrated that miR‐1290 was markedly enriched in LUAD cell‐derived exosomes and could be transferred to macrophages. Notably, the process that hypoxia enhanced miR‐1290 levels was dependent on regulation by HIF‐1α. To gain insight into the effects of miR‐1290 on LUAD progression, we identified that miR‐1290‐deleted exosomes impaired the M2 polarization, which was followed by a decrease in the malignant properties of LUAD cells. These results indicate that hypoxic LUAD cell‐derived exosomal miR‐1290 plays a crucial role in the immune function of macrophages in the TME and is vital for tumor growth and spread.

Numerous studies have implicated suppressor of cytokine signaling 3 (SOCS3) as a key mediator controlling innate and adaptive immunity.^32^, ^33^ SOCS3 is erased or epigenetically downregulated in solid tumors, and functional deficiency has been implicated in tumor progression and metastasis.^34^ Specifically, SOCS3 proteins are most widely known for their role in shaping macrophage polarization.^35^ It was reported that SOCS3 inhibits STAT3 activity, preserving the characteristics of M1‐type macrophages, and without the presence of SOCS3, macrophages are polarized to the M2 phenotype.^36^ Our study showed that miR‐1290 targets the 3'‐UTR of the SOCS3 gene in macrophages and inhibits its expression, resulting in activation of the STAT3 signaling pathway. Accordingly, these data explain how miR‐1290 promotes M2 macrophage polarization through regulation of the SOCS3/STAT3 pathway. The main limitation of this study is lack of analysis on clinical tissue samples. The lung adenocarcinoma samples would be included to further clarify the molecular mechanism in the future.

In summary, our research demonstrated that hypoxic LUAD cell‐derived exosomal miR‐1290 can induce macrophage M2 polarization by activating the SOCS3/STAT3 pathway, which promoted LUAD progression (Figure 6H). These findings suggest that targeting exosomal miR‐1290‐mediated cross talk between tumor cells and TAMs may provide a potential strategy for LUAD immunotherapy.

## AUTHOR CONTRIBUTIONS

**Jiahui Gu:** Formal analysis (equal); investigation (equal); methodology (equal); writing – original draft (equal); writing – review and editing (equal). **Shengrui Yang:** Formal analysis (equal); investigation (equal); methodology (equal); writing – original draft (equal); writing – review and editing (equal). **Xueying Wang:** Investigation (equal); methodology (equal). **Yining Wu:** Investigation (equal); methodology (equal). **Jia Wei:** Investigation (equal); methodology (equal). **Jian Xu:** Conceptualization; formal analysis (equal); funding acquisition; project administration; resources; writing – review and editing (equal).

## CONFLICT OF INTEREST STATEMENT

The authors declare that there are no conflicts of interest.

## Supporting information


Table S1.

## Data Availability

Data available on request from the authors.
