# Supplementary material for: Hypoxic lung adenocarcinoma‐derived exosomal miR‐1290 induces M2 macrophage polarization by targeting SOCS3
Source: Cancer Med. 2023 Apr 20;12(11):12639–52. doi: 10.1002/cam4.5954 (PMC10278512; doi:10.1002/cam4.5954)
Supplement: Supplementary file 1 — Table S1. [file CAM4-12-12639-s001.docx]

**­Table S1.** PCR primers used for qualitative RT-PCR analysis

| **Marker** | **Forward primer（ 5′→ 3′）** | **Reverse primer（ 5′→ 3′）** |
| --- | --- | --- |
| CD80 | CTCTTGGTGCTGGCTGGTC | GCCAGTAGATGCGAGTTTG |
| CD163 | GCTGCAGTGAATTGCACAGAT | CGGGATGAGCGACCTGTT |
| CD206 | GGGTTGCTATCACTCTCTATGC | TTTCTTGTCTGTTGCCGTAGTT |
| MCP | CAGCCAGATGCAATCAATGCC | TGGAATCCTGAACCCACTTCT |
| IL-10 | AGATCTCCGAGATGCCTTCA | CCGTGGAGCAGGTGAAGAAT |
| β-actin | GAGCTACGAGCTGCCTGACG | GTAGTTTCGTGGATGCCACAG |
